# Advances in the study and treatment of glucocorticoid osteoporosis: A review

**DOI:** 10.1097/MD.0000000000042668

**Published:** 2025-05-30

**Authors:** Xingyu Song, Yaheng Zhang, Hongtao Yang, Fujun Xiong, FengFeng Chen

**Affiliations:** a Department of Orthopaedics, Xi’an Daxing Hospital, Xi’an, Shaanxi Province, China.

**Keywords:** glucocorticoids, osteoblast, osteoclast, osteoporosis

## Abstract

In recent years, the widespread application of glucocorticoids in the medical field has led to a notable rise in the incidence of glucocorticoid-induced osteoporosis (GIOP). Currently, glucocorticoid-induced osteoporosis has emerged as the third leading cause of osteoporosis, following senile osteoporosis and postmenopausal osteoporosis. Scientific investigations have demonstrated that glucocorticoids exert a dual-pronged negative impact on bone metabolism. On one hand, glucocorticoids inhibit osteoblasts proliferation, trigger osteoblasts apoptosis, and undermine their osteogenic potential. On the other hand, glucocorticoids enhance osteoclasts proliferation and extend osteoclasts lifespan. Additionally, glucocorticoids can indirectly impede osteogenic differentiation by disrupting the balance of relevant cytokines and compromising the blood supply to bone tissue. This article is a comprehensive review of the latest research findings and treatment advances in the field of glucocorticoid-induced osteoporosis.

## 1. Introduction

Osteoporosis has emerged as a global health issue that cannot be overstated. It has affected approximately 200 million people in China alone, and the World Health Organization has ranked it as the second most significant health threat globally^.[1]^ Clinically, osteoporosis is categorized into 2 main types: primary and secondary. Primary osteoporosis predominantly afflicts the elderly and postmenopausal women. In contrast, secondary osteoporosis stems from a diverse range of factors, including congenital disorders, endocrine dysfunctions, nutritional deficiencies, and the use of specific medications. Glucocorticoid-induced osteoporosis stands as the most prevalent form of secondary osteoporosis, constituting around 20% of all osteoporosis cases.^[2–4]^ The patient population suffering from often includes those with autoimmune diseases such as ankylosing spondylitis and Hashimoto thyroiditis, allergic conditions like purpura, as well as individuals who have undergone organ transplantation.^[5]^ Due to the widespread use of glucocorticoids in the medical field,^[6]^ the issue of glucocorticoid-induced osteoporosis has escalated in severity. As a result, the underlying pathogenesis of glucocorticoid-induced osteoporosis and effective preventive strategies have attracted substantial research interest. This article systematically reviews the latest research findings and therapeutic advancements in the field of glucocorticoid-induced osteoporosis.

## 2. Methods

### 2.1. Search relevant literature

Google search engine was used to search the literature related to glucocorticoid-induced osteoporosis in recent 10 years, and the articles related to mechanism research and drug treatment were screened, read and summarized, and finally completed the writing of the review.

### 2.2. Ethics approval

The review does not involve human or animal studies and therefore does not require ethical approval.

## 3. Results

### 3.1. The process of bone remodeling

Bone remodeling is a normal physiological process involving bone resorption and bone synthesis. Under normal conditions, bone resorption and bone formation are in a state of equilibrium, in which many cytokines, hormones and signaling pathways are involved, with osteoclasts resorbing aged or damaged bone and osteoblasts and osteoclasts being responsible for the formation of new bone.^[7,8]^ When bone homeostasis is disrupted due to disease or application of drugs^[9]^, abnormalities in bone structure or function may occur, leading to disorders of bone metabolism, such as osteosclerosis or osteoporosis. On the 1 hand, osteoblasts and osteoclasts interact with each other to maintain bone microarchitecture and homeostasis in vivo, and osteoblasts and osteoclasts regulate osteoclasts proliferation and differentiation through the secretion of NF-κB ligand (RANKL) and osteoprotegerin receptor activator (OPG)^[10,11]^; on the other hand, the release of activated transforming growth factor-beta (TGF-β) and bone morphogenic protein (BMP) released from the bone matrix after bone resorption also regulate osteoblasts formation.^[12–14]^ In addition, osteoblasts and osteoclasts negatively feedback osteoclasts differentiation by engaging in and synthesizing WNT antagonists, sclerostin (SOST) and Dickkopf 1 (DKK1) to inhibit WNT signaling.^[15]^ Due to osteoblasts interactions, normal in vivo bone remodeling is in a dynamic equilibrium.

### 3.2. Characteristics of the action of glucocorticoid-induced osteoporosis

All forms of osteoporosis exhibit common pathological hallmarks, namely a reduction in total bone mass, bone cortex integrity, and bone matrix quantity. However, glucocorticoid-induced osteoporosis diverges from primary osteoporosis in its underlying pathogenesis.^[16]^ In primary osteoporosis, bone loss predominantly results from an imbalance at the periosteal surface, where bone resorption outpaces bone formation. This imbalance intensifies with aging, leading to a progressive decline in bone mass. Glucocorticoid-induced osteoporosis, conversely, is chiefly characterized by a profound inhibition of bone formation in cancellous bone, accompanied by enhanced bone resorption. Experimental studies on glucocorticoid-induced osteoporosis rats have revealed specific morphological changes, including a decreased percentage of trabecular surface area, thinning of trabecular thickness, and a reduction in trabecular number accompanied by increased trabecular separation.^[17]^ Moreover, glucocorticoids impede the formation of the endosteal surface of bone and stimulate bone resorption. This dual effect contributes to cortical bone thinning and the expansion of the marrow cavity.^[18]^ These distinct pathophysiological mechanisms highlight the need for tailored approaches to the diagnosis, prevention, and treatment of glucocorticoid-induced osteoporosis compared to primary osteoporosis.

### 3.3. Mechanism of action of glucocorticoid-induced osteoporosis

#### 3.3.1. Inhibitory effects of glucocorticoids on osteoblasts and osteoclasts

Osteoblasts primarily originate from bone marrow mesenchymal stem cells (BMSCs). Their key function lies in promoting the formation of the bone cortex, thereby facilitating bone growth. Osteoblasts secrete an array of bone matrix proteins, including osteocalcin and bone growth regulators. These proteins play crucial roles in osteogenesis and the repair of bone microstructural damage. The Wnt/β-catenin signaling pathway is a pivotal regulatory pathway for osteoblasts.^[19]^ At the bone surface, Wnt signaling exerts a dual-fold effect. It not only promotes the differentiation of BMSCs into osteogenic progenitor cells but also inhibits their differentiation towards adipocytes or chondrocytes. In contrast, glucocorticoids disrupt this normal osteogenic regulation. Glucocorticoids drive the differentiation of BMSCs towards adipocytes instead of guiding them towards the osteogenic progenitor cell lineage.^[20]^

The differentiation of osteogenic progenitors into preosteoblasts, and subsequently into osteoblasts, depends on WNT and BMP signaling pathways. These pathways activate the expression of key transcription factors Runx2 and SP7.^[21,22]^ Glucocorticoids disrupt the WNT signaling cascade through multiple mechanisms. Glucocorticoids inhibit WNT signaling by decreasing WNT expression, enhance the expression of WNT antagonists, such as Dkk1, Sost, and secreted crimp-associated protein-1 (sFRP-1), and increase the expression of negative WNT signal regulator Axin-2.^[23,24]^ In parallel, glucocorticoids interfere with BMP signaling. They suppress the expression of BMP-2 and promote the production of BMP antagonists, thereby dampening BMP-mediated osteogenic induction.^[25]^ Moreover, glucocorticoids impede osteoblasts maturation. By suppressing the expression and activity of Runx2, they disrupt the normal progression of osteoblasts differentiation, ultimately contributing to the development of glucocorticoid-induced osteoporosis.

In addition to WNT and BMP, TGF-β is also involved in bone formation.^[26]^ TGF-β promotes regulated osteoblasts differentiation of osteogenic progenitor cells by enhancing WNT signaling.^[27]^ The importance of TGF-β in the regulation of osteoclastogenesis has been experimentally demonstrated by the fact that TGF-β-deficient mice exhibit a significant reduction in bone trabecular density and osteoblasts reduction. According to the relevant literature, glucocorticoids decrease the mRNA level of TGF-β.^[28–30]^

Glucocorticoids are known to induce apoptosis in osteoblasts and osteocytes, with undifferentiated osteoblasts usually dying a few months after formation.^[31]^ Multiple factors, including WNT, TGF-β and estrogen, have been reported to counteract osteoblasts and osteocytes apoptosis.^[32]^ Osteocytes, in contrast to osteoblasts with a lifespan of around 3 months, are long-lived bone cells, surviving for several decades. Osteocytes function as mechanosensory cells. Through their dendritic processes, they can detect microdamage on the bone surface, which may trigger their apoptosis.^[33,34]^ When osteocytes undergo apoptosis, neighboring non-apoptotic osteocytes play a crucial role in the bone remodeling process. They stimulate osteoclastogenesis by releasing interleukin (IL)-6 and soluble IL-6 receptors and secreting receptor activator of nuclear factor-κB ligand (RANKL). These actions attract osteoclasts precursor cells to the site of injury.^[35,36]^ Both in vitro and in vivo studies have demonstrated the pro-apoptotic effects of glucocorticoids on bone cells. In terms of mechanism, glucocorticoids can induce osteoblasts apoptosis by regulating WNT, TGF-β, and IL-6 signaling.^[37]^

### 3.3.2. Glucocorticoids promote osteoclastogenesis and activation. 

It has been shown that glucocorticoids treatment promotes osteoclastogenesis and bone resorption in vitro. RANK is predominantly distributed in bone marrow mesenchymal cells and cells of the osteogenic lineage and binds to its receptor RANKL, which is expressed in osteoblasts progenitors and osteoclasts, thereby inducing osteoclastogenesis and osteoclasts activation.^[38]^ Osteoclast inhibitory factor (OPG) is also expressed in stromal and osteoblasts cell lines and attaches to the RANKL receptor, inhibiting RANK-RANKL interactions, leading to reduced osteoclasts viability and inhibited proliferation.^[39]^ Enhanced bone resorption by glucocorticoids treatment can be explained by the fact that glucocorticoids severely reduces the expression of OPG mRNA, but increases stromal and osteoblastic RANKL mRNA expression, resulting in a significant increase in the ratio of RANKL to OPG.^[40]^ Previous studies have shown that glucocorticoids promotes the expression of miR-17/20a by targeting RANKL, which in turn reinforces the expression of RANKL, while enhancing the osteoclastic activity of osteoblasts.^[41]^ Although osteoblasts strongly express OPG under physiological conditions, glucocorticoid-induced apoptosis of osteoblasts reduces the secretion of OPG to the bone surface, thereby promoting osteoclastogenesis and osteoclasts activation, which itself promotes osteoclastogenesis, osteoclasts activation and bone resorption.^[42]^

It has been reported in the literature that the use of glucocorticoids decreases the number of osteoclast progenitor cells but increases the number of osteoclasts by extending their lifespan.^[43]^ An in vivo mouse transgenic experiment showed that glucocorticoids under the control of the Trap promoter decreased the number of osteoclasts in Hsd11b2 transgenic mice, but extended the lifespan of osteoclasts to enhance bone resorption.^[44]^ Autophagy of osteoclasts is also involved in glucocorticoid-induced bone resorption, and the cellular mechanism of autophagy may ameliorate glucocorticoid-induced bone loss by inhibiting osteoclastogenesis.^[45]^ ROS levels were detected to be significantly upregulated by glucocorticoids in osteoclasts, and n-acetylcysteine (NAC), a ROS scavenger, significantly attenuated the effects of glucocorticoids in inhibiting osteoclasts autophagy and inducing osteoclasts proliferation.^[46,47]^ Thus, ROS are also involved in glucocorticoid-induced osteoclasts proliferation and differentiation.^[48]^

**Figure 1.**
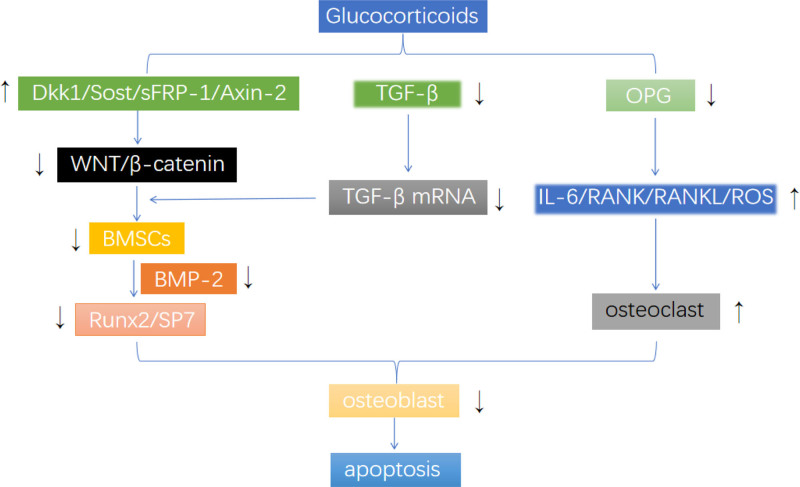
Mechanism of action of glucocorticoids on osteoblasts and osteoclasts.

In summary, glucocorticoids affect osteoblasts and osteoclasts through various mechanisms (Fig. 1), ultimately leading to osteoblasts apoptosis and inducing osteoporosis. However, the specific mechanism of glucocorticoid-induced osteoporosis is still complicated and needs further study.

### 3.4. Progress in the treatment of glucocorticoid-induced osteoporosis

#### 3.4.1. Non-pharmacological treatment

The glucocorticoid-induced osteoporosis International Guidelines provide the following recommendations for the prevention and treatment of non-pharmacological treatment of glucocorticoid-induced osteoporosis: minimise the use and dosage of glucocorticoids as much as possible; all patients are advised to undergo a fall risk assessment and to abstain from smoking and alcohol; exercise that improves strength and balance of the lower limbs, as appropriate, is recommended to reduce the risk of fracture; patients treated with long-term glucocorticoids should be monitored for their daily intake of calcium and vitamin D. The recommended daily intake is 1200 to 1500 mg of calcium and 800 to 1000 IU of vitamin D4.^[49,50]^ Vitamin D4 is a member of the vitamin D family and is used to maintain the body’s calcium and phosphorus balance and promote healthy bone growth.^[51]^ Additional calcium and vitamin D intake is required when patients on long-term glucocorticoids are taking other medications.

#### 3.4.2. Bisphosphonate drugs

Bisphosphonates represent the first class of medications demonstrated to be efficacious in both preventing and treating glucocorticoid-induced osteoporosis.^[52]^ The US Food and Drug Administration has granted approval to risedronate and alendronate for these very purposes.^[53]^ Analysis of drug reports on treatment reveals that bisphosphonates are highly effective in managing glucocorticoids-related vertebral osteoporosis.^[54]^ Their therapeutic potential is further augmented when combined with vitamin D. This combination approach not only addresses the underlying bone loss associated with glucocorticoid-induced osteoporosis but also contributes to enhanced calcium metabolism, providing a more comprehensive strategy for preserving bone health in patients on glucocorticoids therapy.

For patients unable to tolerate oral bisphosphonates, intravenous zoledronic acid or pamidronate serves as an alternative treatment option.^[55]^ Intravenous pamidronate used to be a very effective form of treatment before the advent of intravenous zoledronic acid. However, its use in the treatment of glucocorticoid-induced osteoporosis has declined significantly. Pamidronate requires a long infusion time, and must be administered every 3 months. This frequent dosing regimen not only poses challenges for patients but also limits its practicality in clinical settings. Additionally, pamidronate is contraindicated in patients with renal insufficiency, further restricting its utility. As a result, with the development of alternative medications, pamidronate has gradually faded from clinical use.^[56,57]^ In contrast, intravenous zoledronic acid has overcome many of these limitations, making it a more preferred option for managing glucocorticoid-induced osteoporosis in patients intolerant to oral bisphosphonates.

Zoledronic acid, a third-generation bisphosphonate delivered via intravenous administration, has demonstrated unequivocal efficacy in both preventing and treating glucocorticoid-induced osteoporosis. Multiple experimental studies have validated its effectiveness, and it has received approval for clinical use in the United States.^[58]^ Administered once a year, zoledronic acid circumvents the upper gastrointestinal tract irritation commonly associated with oral bisphosphonates. This advantage has expanded its scope of application, making it a highly attractive option for a broad spectrum of patients at risk of, or already suffering from glucocorticoid-induced osteoporosis.^[59]^ As such, zoledronic acid represents a significant advancement in the pharmacotherapy of glucocorticoid-induced osteoporosis, addressing both efficacy and tolerability concerns.

#### 3.4.3. Teriparatide (parathyroid hormone)

Teriparatide has gained extensive clinical application in the treatment of glucocorticoid-induced osteoporosis. Its anabolic properties, which stimulate osteoblasts and extend their lifespan, play a crucial role in managing glucocorticoid-induced osteoporosis.^[60,61]^ Teriparatide promotes the differentiation of bone progenitor cells into osteoblasts. It also activates existing osteoblasts and safeguards both osteoblasts and osteocytes, effectively countering the detrimental effects of glucocorticoids.^[62]^ This medication exerts a dual, time-dependent influence on bone resorption and formation. When administered intermittently, teriparatide directly enhances osteoblasts activity and indirectly suppresses bone resorption.^[63]^ Research on teriparatide for treating glucocorticoid-induced osteoporosis has shown that it spurs new bone formation and the production of a mineralized bone matrix, significantly elevating serum procollagen type1N-terminal propeptide (P1NP) levels.^[64]^ During long-term teriparatide treatment, levels of bone turnover markers such as P1NP and β-C-terminal telopeptide of type I collagen (β-CTX) remain consistently elevated. This indicates that teriparatide continuously promotes osteogenesis. As a result, it substantially reduces the fracture risk in patients.^[65]^

#### 3.4.4. Denosumab

Denosumab represents the first drug developed to specifically inhibit the osteoclastogenic effects of the receptor activator of nuclear factor-κB ligand (RANKL). It has found extensive clinical application in treating glucocorticoid-induced osteoporosis and a wide range of related bone metabolic disorders.^[66]^ In osteoclast precursor cells, the receptor activator of nuclear factor-κB (RANK) and its ligand, RANKL, are essential for osteoclasts growth, proliferation, activation, and the processes of transcription and translation.^[67,68]^ Osteoprotegerin (OPG), a decoy receptor for RANKL widely distributed across various tissues and organs, can impede RANK protein expression via multiple intracellular signaling pathways. This leads to a substantial inhibition of osteoclasts proliferation and a reduction in osteoclasts activity.^[39,69,70]^ In mouse animal experimental models, the exogenous administration of OPG significantly decreases osteoclasts activity, inhibits bone destruction, and enhances osteoblasts activity.^[71]^ Multiple established animal experimental models have confirmed the pivotal role of OPG in osteoblast-mediated osteogenesis. Mice with in vivo OPG deficiency develop osteoporosis, characterized by a reduction in total bone mass and bone density.^[72–74]^ Conversely, mouse and rodent models with low in vivo RANKL expression and sufficient OPG levels exhibit higher total bone mass, greater bone density, and enhanced bone strength.^[75]^

In a FREEDOM trial, denosumab significantly reduced the risk of fracture in high-risk patients, regardless of age, low BMD, or history of fracture.^[76]^ Denosumab offers distinct advantages in administration. It does not require intravenous injection, and its parenteral delivery method eliminates the risk of upper gastrointestinal intolerance. Moreover, its therapeutic impact on osteoporosis persists for at least 6 months.^[77–79]^ In contrast to bisphosphonates, denosumab can be safely used in patients with chronic kidney disease. This significantly expands the patient population eligible for treatment, enabling more individuals to benefit from its osteoporosis-management capabilities.^[80,81]^

#### 3.4.5. Traditional Chinese Medicine

In traditional Chinese medicine, osteoporosis falls under the category of “bone diseases.” Traditional Chinese medicine theory posits that the kidney stores essence, which generates marrow, and marrow in turn nourishes bones. Traditional Chinese medicine research indicates that deficiency of kidney qi and weakness of the spleen qi are the primary etiological factors contributing to osteoporosis. When treating glucocorticoid-induced osteoporosis, traditional Chinese medicine practitioners select traditional Chinese medicine products that exhibit functions similar to estrogen and androgen, tailored to different traditional Chinese medicine syndrome patterns. These products are typically rich in calcium, phosphorus, potassium, sodium, vitamins, and other essential elements. They effectively inhibit osteoclasts proliferation, stimulate osteoblasts regeneration, and enhance bone formation, demonstrating remarkable efficacy in preventing and treating osteoporosis.^[82–84]^ Through clinical investigations on the prevention and treatment of glucocorticoid-induced osteoporosis with traditional Chinese medicine, researchers have identified several herbs with pronounced preventive and therapeutic effects. Rehmannia glutinosa, Cornus officinalis, Dioscorea opposita, Angelica sinensis, Eucommia ulmoides, Morinda officinalis, and Borneol not only mitigate glucocorticoid-induced osteoporosis but also alleviate the side-effects associated with glucocorticoid medications in patients.^[85–87]^

## 4. Discussion

**Figure 2.**
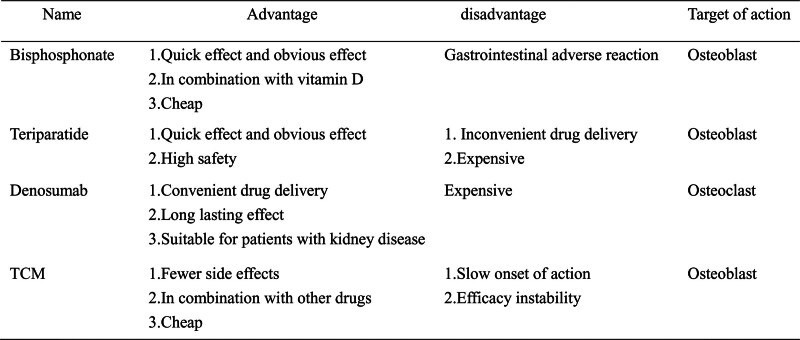
Advantages and disadvantages of drugs for the treatment of glucocorticoid-induced osteoporosis.

In conclusion, in the early stage of glucocorticoids use, patients should be supplemented with an adequate amount of calcium and vitamin D as much as possible to delay the progression of glucocorticoid-induced osteoporosis. Various drugs for the treatment of glucocorticoid-induced osteoporosis have different advantages and disadvantages (Fig. 2). Bisphosphonates have demonstrated remarkable efficacy in the treatment of glucocorticoid-induced osteoporosis. They can be effectively combined with vitamin D to enhance the therapeutic effect. However, their clinical application is restricted by obvious gastrointestinal side effects, which often reduce patients’ compliance. Teriparatide can directly stimulate the proliferation of osteoblasts, accelerate the formation of new bone, and promote the generation of a mineralized bone matrix. Therefore, it can rapidly increase bone mass. Teriparatide has a relatively good safety profile with fewer adverse reactions, making it a promising treatment option. Nevertheless, it requires subcutaneous injection, which is inconvenient for some patients. In addition, its high cost also limits its widespread application. Denosumab is convenient for administration and is not metabolized by the kidneys. This makes it an ideal choice, especially for patients with glucocorticoid-induced osteoporosis complicated by renal insufficiency. However, similar to teriparatide, its high cost restricts its widespread use. Traditional Chinese medicine adopts a personalized treatment approach in the treatment of glucocorticoid-induced osteoporosis, tailoring the prescriptions according to the unique characteristics of each patient. This results in fewer side effects. However, traditional Chinese medicine treatment usually takes a longer time to show results. In addition, differences in the quality of traditional Chinese medicine raw materials may lead to unstable therapeutic effects.

With the widespread clinical use of glucocorticoids, secondary osteoporosis has emerged as an urgent clinical concern. While there are existing guidelines and medications for preventing and treating glucocorticoid-induced osteoporosis, bisphosphonates, teriparatide, denosumab, and traditional Chinese medicine each have their pros and cons. In clinical practice, a comprehensive assessment of patients’ factors is essential when deciding on treatment plans. For most long-term glucocorticoids users at high risk of osteoporosis but without significant renal impairment, like postmenopausal women and the elderly, bisphosphonates are a preferred option. Teriparatide can be used for patients with low bone density and poor response to other treatments. Denosumab is a good choice for those with glucocorticoid-induced osteoporosis and renal insufficiency. Traditional Chinese medicine can be combined with modern drugs to leverage the benefits of integrating traditional and Western medicine.

The mechanism of action of glucocorticoids in inducing osteoporosis is extremely complex, and many detailed molecular mechanisms remain unclear. Research on targeted therapy for signaling pathways related to glucocorticoid-induced osteoporosis is underway, and new treatment approaches may emerge in the future. In-depth research on the mechanism of action of glucocorticoid-induced osteoporosis and the search for effective therapeutic drugs will significantly reduce the incidence of glucocorticoid-induced osteoporosis and thereby decrease the number of patients with this condition. We believe that with the continuous in-depth experimental research, the pathogenesis of glucocorticoid-induced osteoporosis will be better understood and recognized at the molecular and genetic levels. This will lead to the introduction of new means for the prevention and treatment of glucocorticoid-induced osteoporosis, ultimately laying the foundation for the active prevention and treatment of this disease.

## Author contributions

**Conceptualization:** Xingyu Song, Yaheng Zhang, Hongtao Yang, Fujun Xiong, FengFeng Chen.

**Data curation:** Yaheng Zhang, Hongtao Yang.

**Methodology:** Hongtao Yang.

**Writing – original draft:** Xingyu Song, Fujun Xiong, FengFeng Chen.

**Writing – review & editing:** Xingyu Song, Fujun Xiong, FengFeng Chen.
